# Cost-effectiveness analysis of combined immunotherapy regimens for non-squamous non-small lung cancer after tyrosine kinase inhibitors resistance

**DOI:** 10.3389/fpubh.2026.1860791

**Published:** 2026-07-23

**Authors:** Mengmeng Teng, Bo Yang, Ruikang Zhang, Fuhong Qin, Jianwei Ji

**Affiliations:** 1 Department of Pharmacy, The Second Affiliated Hospital of Zhengzhou University, Zhengzhou, China; 2 Department of Epidemiology and Health Statistics, School of Public Health, Fudan University, Shanghai, China

**Keywords:** cost-effectiveness, immunotherapy, ivonescimab, non-squamous non-small cell lung cancer, tyrosine kinase inhibitor resistance

## Abstract

**Background:**

Immunotherapy combined with anti-angiogenic agents and chemotherapy has emerged as a novel therapeutic option for non-squamous non-small cell lung cancer (non-sqNSCLC) after resistance to tyrosine kinase inhibitors (TKIs). This study evaluated the efficacy, safety, and cost-effectiveness of these regimens in patients with TKI-resistant non-sqNSCLC in China.

**Methods:**

A network meta-analysis was performed to compare the efficacy and safety of ivonescimab plus chemotherapy (Ivon-Chem), sintilimab plus bevacizumab and chemotherapy (Sint-Beva-Chem), and atezolizumab plus bevacizumab and chemotherapy (Atez-Beva-Chem). A Markov model was constructed to assess the cost-effectiveness of Ivon-Chem, Sint-Beva-Chem, Atez-Beva-Chem, and chemotherapy until the survival probability fell below 1% in any regimen. The incremental cost-effectiveness ratio (ICER) and net monetary benefit (NMB) were estimated using a willingness-to-pay (WTP) threshold of $41,859. Sensitivity analyses and scenario simulations were also performed.

**Results:**

Ivon-Chem, Sint-Beva-Chem, and Atez-Beva-Chem improved efficacy but were associated with poorer safety profiles than chemotherapy. Compared with chemotherapy, Ivon-Chem, Sint-Beva-Chem, and Atez-Beva-Chem were not cost-effective, with ICERs of $61,882.3/QALY, $68,657.0/QALY, and $188,820.5/QALY, respectively. Moreover, the NMBs of Ivon-Chem, Sint-Beva-Chem and Atez-Beva-Chem were all negative, indicating that none of these regimens was economically acceptable. Scenario analyses indicated that, compared with chemotherapy, Ivon-Chem would become cost-effective if the price of ivonescimab were reduced by at least 47.2%. Similarly, Sint-Beva-Chem and Atez-Beva-Chem would achieve cost-effectiveness following price reductions of 59.3% for both sintilimab and bevacizumab, and 84.4% for both atezolizumab and bevacizumab, respectively.

**Conclusion:**

These immunotherapy-based regimens provided significant survival benefits in patients with TKI-resistant non-sqNSCLC. However, from the perspective of the Chinese healthcare system, Ivon-Chem, Sint-Beva-Chem, and Atez-Beva-Chem were not cost-effective. Substantial price reductions would be required to improve their economic viability.

## 1 Introduction

Non-small cell lung cancer (NSCLC) is one of the leading causes of cancer-related morbidity and mortality worldwide (1, 2). The treatment of NSCLC primarily involves systemic therapies, including chemotherapy, targeted therapy, and immunotherapy (3). Non-squamous NSCLC (non-sqNSCLC) accounts for approximately 78% of NSCLC cases, and genetic alterations, such as epidermal growth factor receptor (EGFR) mutations and anaplastic lymphoma kinase (ALK) rearrangements, occur in more than 50% of non-sqNSCLC cases (1, 2). In patients with driver mutation-positive non-sqNSCLC, EGFR or ALK-targeted tyrosine kinase inhibitors (TKIs) have demonstrated significantly greater efficacy than chemotherapy and have become the standard first-line treatment (4, 5). In the OPTIMAL study, TKI therapy significantly improved median progression-free survival (mPFS; 13.1 vs. 4.6 months), although overall survival (OS) was similar to that with chemotherapy (6, 7). However, most patients experience disease progression after 9–13 months of treatment with first-, second-, or third-generation TKIs (8, 9). Drug resistance remains a major challenge, and immunotherapy has emerged as a promising therapeutic option after the development of resistance to TKIs.

The combination of immune checkpoint inhibitors (ICIs), anti-angiogenic agents, and chemotherapy has emerged as a novel strategy for NSCLC with genetic alterations. In the ATTLAS trial, the addition of atezolizumab and bevacizumab to chemotherapy (Atez-Beva-Chem) extended mPFS to 8.5 months, nearly 3 months longer than chemotherapy alone (5.6 months), in patients with EGFR-sensitizing mutations or ALK alterations (10). Comparable benefits have been observed with sintilimab plus bevacizumab and chemotherapy (Sint-Beva-Chem) in patients with non-sqNSCLC after TKI failure (11, 12). More recently, ivonescimab, a dual immune checkpoint and anti-angiogenic bispecific antibody, was approved for the treatment of non-sqNSCLC. The objective response rate of ivonescimab plus chemotherapy (Ivon-Chem) was 50.6%, compared with 35.4% in the chemotherapy group (12, 13). Based on these clinical studies, the current Chinese Society of Clinical Oncology guidelines recommend Atez-Beva-Chem, Sint-Beva-Chem, and Ivon-Chem as first-line therapeutic options for non-sqNSCLC with genetic alterations who have progressed after TKI therapy (3, 14). Although these regimens significantly prolong survival compared with chemotherapy alone, their costs are substantially higher. Previous cost-effectiveness studies suggested that, under China’s economic conditions, these regimens may not be economically acceptable compared with chemotherapy (15, 16). However, there is a lack of economic evaluations of Atez-Beva-Chem from the ATTLAS trial. The efficacy, safety, and cost-effectiveness of immunotherapy plus anti-angiogenic agents and chemotherapy for the treatment of non-sqNSCLC with genetic alterations still require further investigation.

Network meta-analysis has become a useful approach for indirectly comparing the efficacy and safety of immunotherapy plus anti-angiogenic agents and chemotherapy, thereby providing critical clinical evidence (17, 18). This study aimed to evaluate the efficacy, safety, and cost-effectiveness of immunotherapy plus anti-angiogenic agent regimens and chemotherapy in non-sqNSCLC with genetic alterations following TKI resistance in China, thereby providing valuable guidance for clinical decision-making.

## 2 Methods

### 2.1 Literature search and filters

We performed a comprehensive literature search across PubMed, Web of Science, Embase, and Cochrane Library for eligible publications, published up to December 2025. The search terms were “ivonescimab,” “atezolizumab,” “sintilimab,” and “bevacizumab” in “non-squamous non-small cell lung cancer with genetic alterations.” Studies were included if they involved non-sqNSCLC with genetic alterations, progression following TKIs therapy, immunotherapy with bevacizumab as the intervention, and chemotherapy as the control. The studies were excluded if participants were younger than 18 years, the study was not a randomized controlled trial (RCT), efficacy data were unavailable, or the study was a duplicate publication. Details of search and filters are shown in Supplementary Table S1 and Supplementary Figure S1. The methodological quality and risk of bias were assessed according to the Cochrane Handbook (Supplementary Figure S2). The protocol was prospectively registered with PROSPERO (CRD420251250541). As this study did not involve primary or individual participant data, ethical approval was not required.

### 2.2 Efficacy and safety in network meta-analysis

Data were extracted from clinical trials and recent meeting abstracts by TMM, YB, and ZRK. The network meta-analysis was conducted using R software (version 4.4.2) with the *“netmeta”* package. Hazard ratios (HRs) with 95% confidence intervals (CIs) were calculated for OS and PFS, and odds ratios (ORs) with 95%CIs were calculated for adverse events (AEs) and severe AEs (grade ≥3, sAEs). Heterogeneity across included trials and publication bias were also assessed. The surface under the cumulative ranking curve (SUCRA) was used to assess and rank the relative efficacy and safety of the four strategies.

### 2.3 Model structure

**Figure 1:**
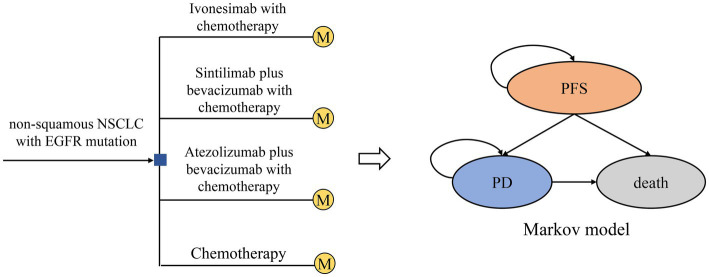
The Markov model. NSCLC, non-small cell lung cancer; EGFR, epidermal growth factor receptor; PFS, progression-free survival; PD, progression disease.

The Markov model was constructed using R (version 4.2.2) with the *“heemod”* package to evaluate the cost-effectiveness of treatment strategies in patients with genetically altered non-sqNSCLC after the development of resistance to TKIs (19). The economic evaluation included four strategies: Ivon-Chem, Sint-Beva-Chem, Atez-Beva-Chem, and chemotherapy. The Markov model consisted of three health states (Figure 1): PFS, progressive disease (PD), and death. All patients initially entered the PFS state, and subsequently remained progression-free or transitioned to PD or death. The probability of transition from PFS to death was based on the natural mortality rate in China (0.7%). Patients in the PD state either remained in PD or transitioned to death, and death was an absorbing state. The cycle length was set at 3 weeks in the Markov model to reflect the treatment schedules, and time horizon was maintained until the survival rate fell below 1% in any strategy.

### 2.4 Survival data reconstruction

State transition probabilities were calculated from PFS and OS data from RCTs (10–13, 20). The numbers of patients at risk were extracted, and Kaplan–Meier (KM) curves for OS and PFS were digitized using WebPlotDigitizer.^1^ Individual patient data (IPD) were reconstructed with the *“IPDfromKM”* package, and the KM curves were plotted using the reconstructed IPD (Supplementary Figure S3). The reconstructed IPD were subsequently fitted to parametric survival models, including Weibull, exponential, log-logistic, lognormal, gamma, generalized gamma, and Gompertz distributions using the *“flexsurv”* package (21). The PFS and OS data of chemotherapy were derived from reconstructed IPD obtained from the three RCTs (10–13, 20). The best-fitting distributions were identified according to Akaike information criterion and Bayesian information criterion (Supplementary Table S2; Supplementary Figure S4), and were used to extrapolate survival beyond the trial follow-up period. These fitted survival functions were subsequently applied to estimate transition probabilities in the Markov model.

Cycle-specific transition probabilities were calculated from the fitted PFS and OS curves. The probability of transition from the PFS state to death (P_PFS → Death_) was estimated using natural mortality. The transition from the PD state to death (P_PD → Death_) was estimated based on the reduction in PD populations derived from OS and PFS curves between cycles. The probability of progression (P_PFS → PD_) was calculated from the decrease in PFS survival rates between two adjacent cycles, excluding P_PFS → Death_ and P_PD → Death._ Then the probability of remaining PFS (P_PFS → PFS_) was calculated as P_PFS → PFS_ = 1 − P_PFS → PD_ − P_PFS → Death._ The probability of remaining in the PD state was calculated in PD state populations as P_PD → PD_ = 1 − P_PD → Death_. A schematic representation of the state transitions is shown in Supplementary Figure S5.

### 2.5 Cost-effectiveness analyses

This health economic evaluation was conducted from the perspective of the Chinese healthcare system and included direct costs. Drug costs were obtained from a local database,^2^ while treatment costs for sAEs, follow-up costs (laboratory tests, radiologic tests, and administration), best supportive care, subsequent therapy cost, and terminal care costs were obtained from literature (15, 22). Utility values for PFS and PD states, as well as disutility values of sAEs, were also derived from literature (Table 1) (16, 23). The cost and disutility values for sAEs were derived from the ORIENT-31 (16), and those for Ivon-Chem, Atez-Beva-Chem and chemotherapy groups were adjusted according to the ratio of the incidence of sAEs relative to that of Sint-Beva-Chem (10–13, 20). These cost and utility values were discounted at an annual rate of 5%, with a sensitivity range of 0–8%.

**Table 1:** Clinical data, cost and utility in Markov model.

Variable	Value	Range	Distribution	References
Clinical parameters				
Survival model for ivon+chem				(13, 20)
Overall survival	mu = 3.2328, sigma = 0.8892		log-normal	
Progression-free survival	shape = 2.2613, scale = 10.9696		log-logistic	
Survival model for atez+beva+chem				(10)
Overall survival	mu = 3.3851, sigma = 0.8979		log-normal	
Progression-free survival	mu = 2.5921, sigma = 0.7500		log-normal	
Survival model for sint+beva+chem				(11, 12)
Overall survival	sigma = 0.8271, k = 2.2401, theta = 15.4727		Gamma	
Progression-free survival	mu = 2.4189, sigma = 0.8271		log-normal	
Survival model for chem				(10–13, 20)
Overall survival	shape = 1.9076, scale = 17.0675		log-logistic	
Progression-free survival	mu = 1.5898, sigma = 0.8290		log-normal	
Risk for AEs (above grade 3)				
Ivon+chem group	61.5%		Beta	(13, 20)
Sint+beva+chem group	55.7%		Beta	(11, 12)
Atez+beva+chem group	35.1%		Beta	(10)
Chem group	42.8%		Beta	(10–13, 20)
Health utility				
Utility of progression-free survival	0.804	0.64–0.96	Beta	(23)
Utility of progression disease	0.321	0.26–0.39	Beta	(23)
Disutility of AEs (≥ grade 3)^*^	0.00616	0.00462–0.00770	Beta	(16)
Cost ($)				
Ivonescimab (100 mg)	104.0	83.2–124.8	Gamma	Local
Atezolizumab (1,200 mg)	4635.9	3708.7–5563.1	Gamma	Local
sintilimab (200 mg)	152.6	122.1–183.1	Gamma	Local
Bevacizumab (100 mg)	152.0	121.6–182.4	Gamma	Local
IBI305 (100 mg)	164.0	131.2–196.8	Gamma	Local
Pemetrexed (500 mg)	84.4	67.5–101.3	Gamma	Local
Carboplatin (150 mg)	17.0	13.6–20.4	Gamma	Local
Cisplatin per (10 mg)	3.1	2.5–3.7	Gamma	Local
Paclitaxel (100 mg)	14.5	11.6–17.4	Gamma	Local
Costs of AEs (≥ grade 3) ^*^	107.5	80.6–134.3	Gamma	(16)
Follow-up (per cycle)	55.6	44.5–66.7	Gamma	(22)
Best supportive care (per cycle)	339	271.2–406.8	Gamma	(15)
Subsequent therapy cost (per cycle)	854.1	683.2–1024.9	Gamma	(22)
Terminal care per patient	2,475	1980–2,970	Gamma	(15)
Creatinine clearance rate (mL/min)	80	64–96	Normal	(22)
Body surface area (m^2^)	1.72	1.38–2.06	Normal	(22)
Weight (kg)	64	51.2–76.8	Normal	(22)

^*^Disutility and cost of AEs from ORIENT-31, ivon+chem, ivonescimab with chemotherapy; atez+beva+chem, atezolizumab plus bevacizumab with chemotherapy; sint+beva+chem, sintilimab plus bevacizumab with chemotherapy; AEs, adverse events.

Total costs, quality-adjusted life years (QALYs), and life years (LYs) were calculated in the Markov model. The willingness-to-pay (WTP) threshold for China was $41,859 (three times the GDP per capita in 2025). Cost-effectiveness was expressed as incremental cost-effectiveness ratio (ICER) in QALYs, where ICER = (Cost_1_ − Cost_2_)/(QALY_1_ − QALY_2_). An ICER below the WTP threshold ($41,859/QALY) indicated that the strategy was considered cost-effective compared with the reference strategy. In addition, net monetary benefit (NMB) was calculated for each strategy using the formula: QALYs × WTP threshold − Cost. A positive NMB (NMB > 0) indicated that the strategy was considered an economically acceptable option at the WTP threshold ($41,859). Furthermore, ICERs based on LYs gained were evaluated as a supplementary analysis. An ICER per LY gained below the WTP threshold indicated that the intervention could be considered as a potentially acceptable therapeutic option compared with the reference strategy.

### 2.6 Sensitivity analysis

In the sensitivity analysis, various parameters, including costs and utilities, were incorporated for evaluation. Deterministic one-way sensitivity analysis (DSA) was applied to examine the impact of changes in individual variables on overall model outcomes. The results of DSA were typically illustrated using tornado diagrams, which quantified the influence of variation in each variable on the ICER compared with chemotherapy. Probabilistic sensitivity analysis (PSA) was conducted based on the distributions of variables, using 2,000 Monte Carlo simulations. The PSA outcomes were presented as scatter plot and cost-effectiveness acceptability curve (CEAC), which were used to characterize decision uncertainty and evaluate the economic acceptability of each treatment strategy. Across a WTP threshold range of $0–$200,000 per QALY gained, the probability of a strategy being the economically acceptable option was defined as the proportion of simulations in which the strategy generated the highest NMB among all competing strategies. The scatter plot displayed the distribution of costs and QALYs generated in each PSA iteration. The CEAC illustrated the probability that each treatment strategy would generate the highest NMB and therefore be considered the economically preferred option at a given WTP threshold.

### 2.7 Scenario simulations

Additionally, scenario simulations were performed to assess the impact of hypothetical price reductions for ivonescimab, sintilimab, atezolizumab, and bevacizumab, ranging from 0 to 100%. These simulations were used to evaluate ICERs using chemotherapy as the reference strategy and the NMBs of treatment strategies under varying price scenarios. The price-reduction threshold at which the ICER fell below the WTP threshold ($41,859) represented the level at which the treatment strategy became cost-effective compared with chemotherapy. In contrast, the price-reduction threshold at which the NMB became positive indicated that the strategy generated positive net economic value and was therefore economically acceptable at the WTP threshold ($41,859). Furthermore, the total costs during the PFS state were adjusted based on current treatment costs to achieve NMB = 0, ensuring that treatment costs during the PFS period remained within acceptable limits.

### 2.8 Statistical analysis

Statistical analyses and plots were performed in RStudio (R version 4.3.2) and Microsoft Excel (version 2021). Reporting of this study followed the Health Economic Evaluation Reporting Standards 2022 (CHEERS 2022) statement (Supplementary Table S3).

## 3 Results

### 3.1 Network meta-analysis in efficacy and safety

**Figure 2:**
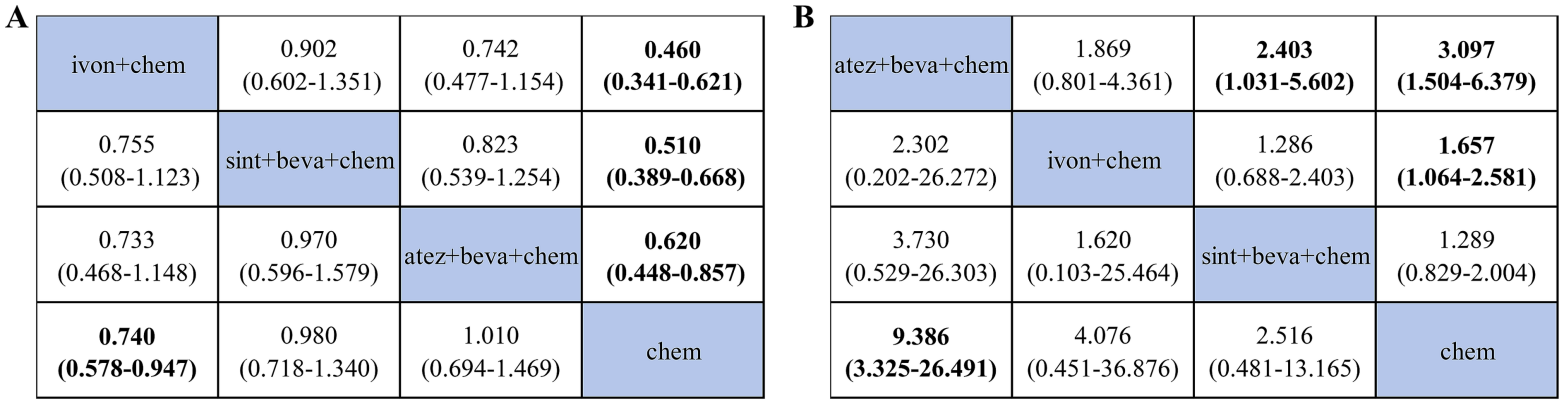
Efficacy and safety based on network meta-analysis. **(A)** Overall survival (left-below) and progression-free survival (right-up). **(B)** Adverse events (left-below) and grade 3 or higher adverse events (right-up). Ivon+chem, ivonescimab with chemotherapy; sint+beva+chem, sintilimab plus bevacizumab with chemotherapy; atez+beva+chem, atezolizumab plus bevacizumab with chemotherapy; chem, chemotherapy.

Based on the predefined inclusion and exclusion criteria, three phase III RCTs were ultimately included in the analysis (Supplementary Figure S1): HARMONi-A (13, 20), ORIENT-31 (11, 12), and ATTLAS (10). Detailed study characteristics are presented in Supplementary Table S4, and the included studies evaluated four treatment regimens: Ivon-Chem, Sint-Beva-Chem, Atez-Beva-Chem, and chemotherapy alone. Although the network lacked closed loop (Supplementary Figure S6A), no significant heterogeneity or publication bias was observed (Supplementary Table S5). The network meta-analysis showed that, compared with chemotherapy alone, significant improvements in PFS were observed with Ivon-Chem (HR: 0.460, *p* < 0.05), Sint-Beva-Chem (HR: 0.510, *p* < 0.05), and Atez-Beva-Chem (HR: 0.620, *p* < 0.05) (Figure 2A). However, for OS, only Ivon-Chem demonstrated a statistically significant improvement (HR: 0.740, *p* < 0.05). Regarding safety, the risk of sAEs was markedly higher in Atez-Beva-Chem than in Sint-Beva-Chem and chemotherapy alone, with increases of 2.4-fold and 3.1-fold, respectively (*p* < 0.05) (Figure 2B). Similarly, Ivon-Chem was associated with a significantly elevated risk of sAEs, with a 1.6-fold higher risk than chemotherapy alone (*p* < 0.05). According to the SUCRA rankings, Ivon-Chem ranked highest for efficacy, whereas chemotherapy alone demonstrated the most favorable safety profile (Supplementary Figure S6A; Supplementary Table S6).

### 3.2 Base-case analysis

The Markov model was used to simulate patient transitions among the PFS, PD, and death states over a 5-year time horizon (85 cycles). Table 2 summarizes the base-case analysis. Compared with chemotherapy, the ICERs for Ivon-Chem, Sint-Beva-Chem, and Atez-Beva-Chem were $61,882.3/QALY, $68,657.0/QALY, and $188,820.5/QALY, respectively, all exceeding the WTP threshold ($41,859/QALY). Based on the ICER results, none of the three regimens was identified as cost-effective at the predefined WTP threshold. Furthermore, the NMBs for Ivon-Chem (−$3,215.6), Sint-Beva-Chem (−$39,943.6) and Atez-Beva-Chem (−$80,890.5) were negative, indicating that none was economically acceptable. In contrast, chemotherapy was the only strategy with a positive NMB ($5,772.9), suggesting that it was the only economically acceptable option under the current WTP threshold. Moreover, the ICER analysis based on LYs gained suggested that Sint-Beva-Chem may represent a potentially acceptable therapeutic option compared with chemotherapy, Ivon-Chem, and Atez-Beva-Chem (Table 3).

**Table 2:** Base case analysis results.

Regimens	Cost ($)	QALYs	Reference	Cost difference ($)	QALYs difference	ICER ($/QALY)
Chem	16,647.8	0.536				
Ivon+chem	44,426.9	0.985	Chem	27,779.1	0.449	61,882.3
Sint+beva+chem	56,913.9	1.122	Chem	40,266.1	0.586	68,657.0
			Ivon+chem	12,487.0	0.138	90,761.8
Atez+beva+chem	127,995.6	1.125	Chem	111,347.8	0.590	188,820.5
			Ivon+chem	83,568.7	0.141	593,529.4
			Sint+beva+chem	71,081.7	0.003	22,078,484.2

Ivon+chem, ivonescimab with chemotherapy; sint+beva+chem, sintilimab plus bevacizumab with chemotherapy; atez+beva+chem, atezolizumab plus bevacizumab with chemotherapy; chem, chemotherapy. QALYs, quality-adjusted life years; ICER, incremental cost-effectiveness ratio; NMB, Net monetary benefit.

**Table 3:** The incremental cost-effectiveness ratio in life years.

Regimens	Cost ($)	LYs	Reference	Cost difference ($)	LYs difference	ICER ($/LY)
Chem	16,647.8	1.145				
Ivon+chem	44,426.9	1.981	Chem	27,779.1	0.837	**33,207.1**
Sint+beva+chem	56,913.9	2.377	Chem	40,266.1	1.232	**32,688.7**
			Ivon+chem	12,487.0	0.395	**31,591.7**
Atez+beva+chem	127,995.6	2.218	Chem	111,347.8	1.073	103,743.5
			Ivon+chem	83,568.7	0.237	352,971.0
			Sint+beva+chem	71,081.7	−0.159	**Undominated**

Bold values denote cost-effective regimens compared to the reference at the current willing-to-pay threshold ($41,859).

ivon+chem, ivonescimab with chemotherapy; sint+beva+chem, sintilimab plus bevacizumab with chemotherapy; atez+beva+chem, atezolizumab plus bevacizumab with chemotherapy; chem, chemotherapy. LYs, life years; ICER, incremental cost-effectiveness ratio.

### 3.3 Sensitivity analysis

**Figure 3:**
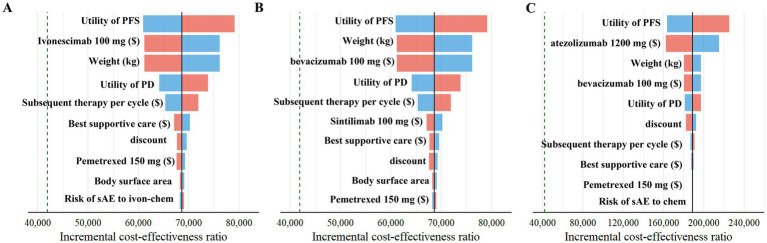
Tornado diagram of one-way sensitivity analyses. **(A)** Ivonescimab with chemotherapy. **(B)** Sintilimab plus bevacizumab with chemotherapy. **(C)** Atezolizumab plus bevacizumab with chemotherapy. PFS, progression-free survival; PD, progression disease.

The DSA was performed for ICERs compared with chemotherapy (Figure 3). The utility of the PFS state, drug cost, and subsequent therapy cost were the most influential parameters for Ivon-Chem, Sint-Beva-Chem, and Atez-Beva-Chem. Despite variations in these parameters, the ICERs consistently remained above the predefined WTP threshold, indicating that no significant change affected the overall conclusion. These findings demonstrated that the Markov model for evaluating cost-effectiveness was robust, and that the base-case conclusions remained stable across a wide range of plausible input values.

**Figure 4:**
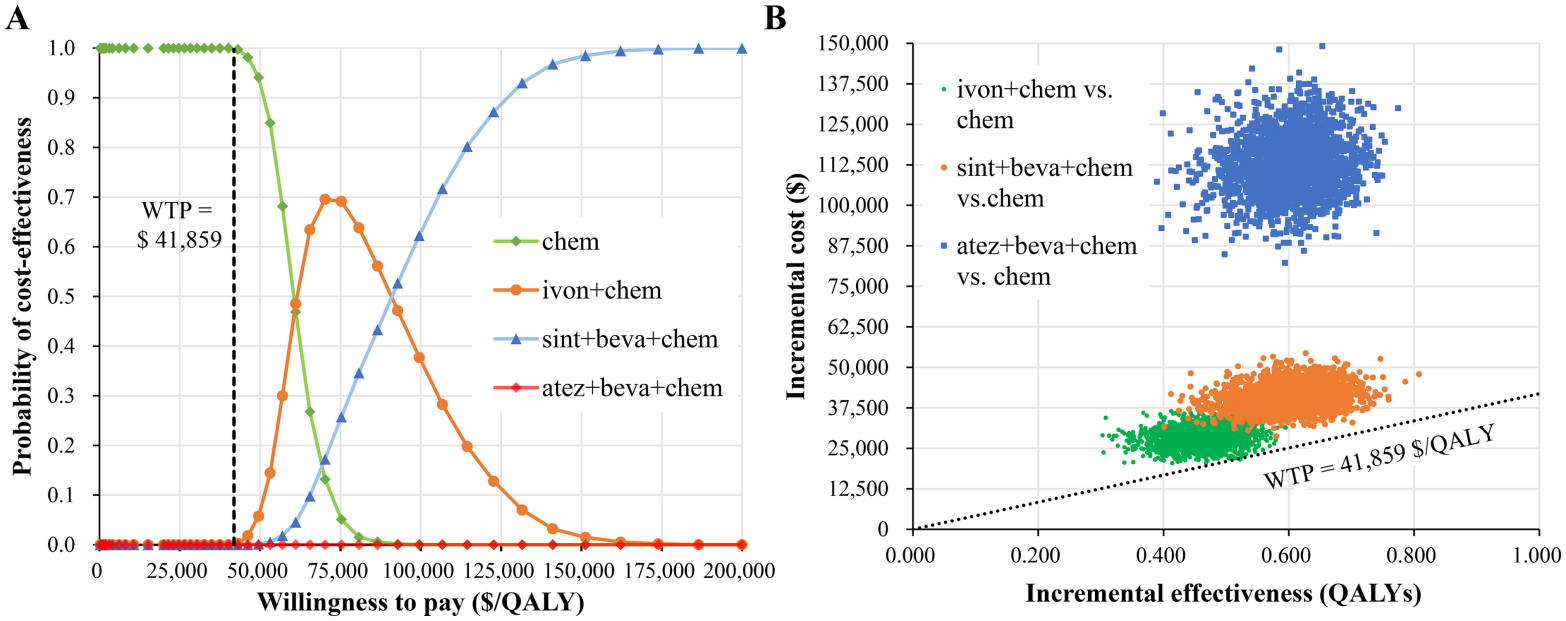
Probabilistic sensitivity analysis. **(A)** Acceptability curves of cost-effectiveness. **(B)** Incremental cost-effectiveness ratio plane. Ivon+chem, ivonescimab with chemotherapy; sint+beva+chem, sintilimab plus bevacizumab with chemotherapy; atez+beva+chem, atezolizumab plus bevacizumab with chemotherapy.

The PSA was conducted using 2,000 Monte Carlo simulations to assess parameter uncertainty. The CEAC and scatter plot demonstrated that chemotherapy had a 100% probability of generating the highest NMB among all four treatment strategies at a WTP threshold of $41,859 (Figure 4), making it the economically preferred strategy. At a WTP threshold of $75,000, the probabilities of generating the highest NMB were 70% for Ivon-Chem and 25% for Sint-Beva-Chem. When the WTP threshold exceeded $90,000, Sint-Beva-Chem had the greatest probability of generating the highest NMB. In contrast, Atez-Beva-Chem consistently exhibited a low probability of generating the highest NMB across the evaluated WTP range.

### 3.4 Scenario analysis: impact of drug price reduction

**Figure 5:**
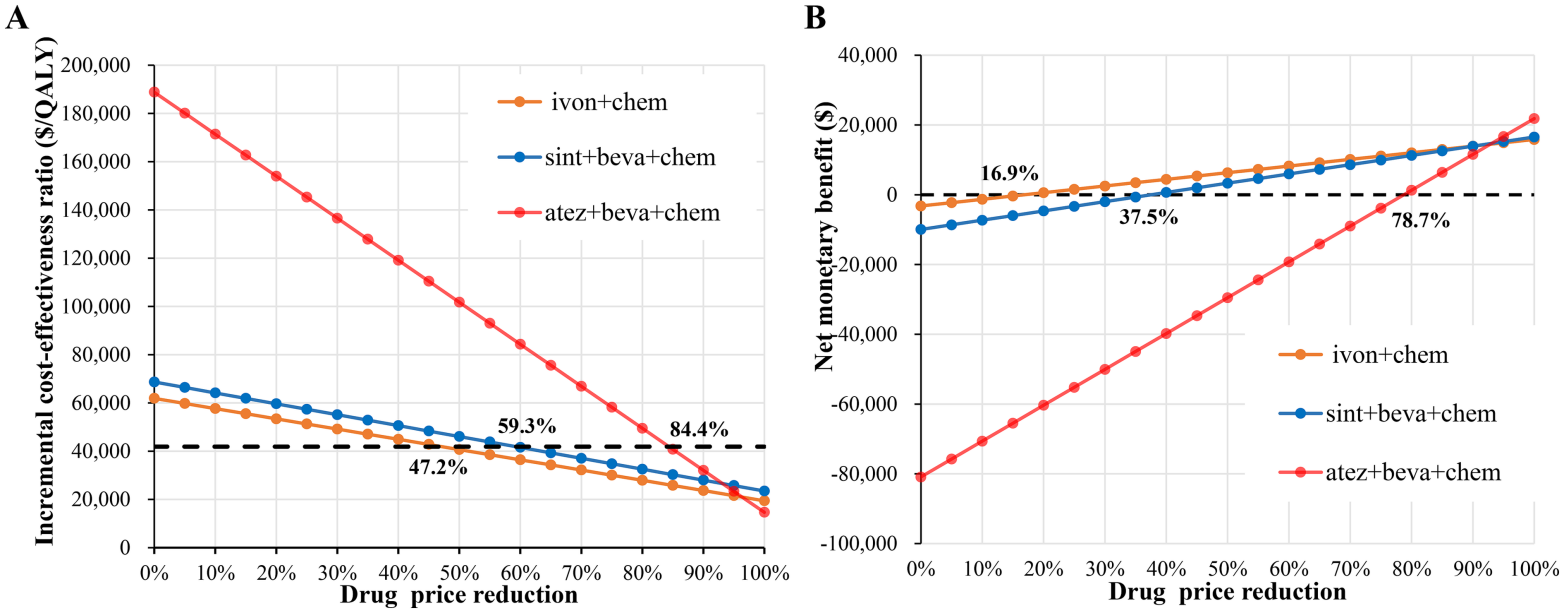
Scenario analysis exploring drug price reduction. **(A)** Incremental cost-effectiveness ratio. **(B)** Net monetary benefit. Ivon+chem, ivonescimab with chemotherapy; sint+beva+chem, sintilimab plus bevacizumab with chemotherapy; atez+beva+chem, atezolizumab plus bevacizumab with chemotherapy.

The scenario analyses based on ICER revealed price-reduction thresholds at which each regimen could become cost-effective compared with chemotherapy (Figure 5): a 47.2% reduction in the price of ivonescimab for Ivon-Chem, a 59.3% reduction in the price of both sintilimab and bevacizumab for Sint-Beva-Chem, and an 84.4% reduction in the price of both atezolizumab and bevacizumab for Atez-Beva-Chem. In contrast, the NMB analyses indicated the price-reduction thresholds at which each regimen could become economically acceptable (Figure 5): a 16.9% reduction in the price of ivonescimab for Ivon-Chem, a 37.5% reduction in the price of both sintilimab and bevacizumab for Sint-Beva-Chem, and an 78.7% reduction in the price of both atezolizumab and bevacizumab for Atez-Beva-Chem. Further NMB-based analyses suggested that these regimens could become economically acceptable when PFS-related treatment costs were reduced (Supplementary Figure S7).

## 4 Discussion

The advent of immunotherapy has profoundly reshaped the treatment landscape for patients with non-sqNSCLC who have experienced disease progression following TKI therapy. In China, Ivon-Chem, Sint-Beva-Chem, and Atez-Beva-Chem have been adopted as therapeutic options for patients with non-sqNSCLC after the development of resistance to TKIs. Three RCTs, including HARMONi-A, ORIENT-31, and ATTLAS, have demonstrated a survival advantage of Ivon-Chem, Sint-Beva-Chem, and Atez-Beva-Chem over chemotherapy in TKI-resistant non-sqNSCLC (10, 11, 20). However, the substantial economic burden associated with these high-cost combination regimens has made the optimal balance among efficacy, safety, and cost-effectiveness in the management of non-sqNSCLC unclear. This study represents a comprehensive effort to systematically compare the profiles of Ivon-Chem, Sint-Beva-Chem, Atez-Beva-Chem, and chemotherapy through an integrated assessment of clinical efficacy, safety, and economic outcomes.

The results of the network meta-analysis indicated that Ivon-Chem, Sint-Beva-Chem, and Atez-Beva-Chem significantly prolonged PFS compared with chemotherapy. However, only the Ivon-Chem regimen demonstrated a significant improvement in OS, suggesting its potential for greater clinical benefit. Treatment rankings based on SUCRA further supported the favorable profile of Ivon-Chem for both OS and PFS. Notably, compared with chemotherapy alone, both Atez-Beva-Chem and Ivon-Chem were associated with a significantly increased incidence of sAEs. Moreover, the incidence of sAEs was higher with Atez-Beva-Chem than with Sint-Beva-Chem. Combination regimens integrating immunotherapy, anti-angiogenic agents, and chemotherapy have emerged as a major focus in lung cancer treatment because of their superior therapeutic efficacy (24, 25). These benefits may be attributed to synergistic effects involving anti-angiogenesis, immune activation, and cytotoxic activity, including inhibition of angiogenesis to remodel the tumor microenvironment, thereby enhancing the ability of ICIs to recognize and eliminate tumor cells, together with the direct cytotoxic effects of chemotherapy (26). However, the increased toxicity associated with these regimens cannot be overlooked. Although predictive biomarkers may be important for explaining differential treatment responses and guiding personalized strategies, validated biomarkers remain unavailable. Future studies should prioritize the discovery and validation of such markers to enable treatment approaches that optimize efficacy and safety.

This study employed a Markov model for economic evaluation. Markov models, which can describe disease progression in detail through dynamic transition probabilities, have been widely applied in economic assessments of cancer (17, 19). To calculate dynamic transitions, we reconstructed individual patient data and applied multiple methods to fit survival curves, selecting the best-fitting distributions (21). All individual patient data were derived from the most recent data available from each study to incorporate the longest possible follow-up duration and minimize extrapolation bias during curve extension. Furthermore, both discounting and half-cycle correction were applied within the model to mitigate the influence of the study time horizon on the results (27).

At the prespecified WTP threshold of $41,859/QALY, none of the Ivon-Chem, Sint-Beva-Chem, or Atez-Beva-Chem regimens was cost-effective compared with chemotherapy (ICER > WTP). Although the three regimens showed similar clinical benefits, the ICERs for Sint-Beva-Chem and Atez-Beva-Chem compared with Ivon-Chem were substantially higher than the WTP threshold, indicating that these regimens were not cost-effective at the predefined WTP threshold. Consistent with these findings, the ICERs for Ivon-Chem and Sint-Beva-Chem compared with chemotherapy were $270,507.7/QALY and $53,266.3/QALY, respectively (15, 16). In the Atez-Beva-Chem regimen, the cost of atezolizumab is considerably higher than that of ivonescimab and sintilimab, and unless it offers substantially higher utility, it is unlikely to be cost-effective. This finding is supported by the PSA results, which showed that Atez-Beva-Chem is unlikely to be the economically acceptable strategy across a wide range of WTP values. To evaluate the economic acceptability of each strategy, we conducted analyses based on NMB. Unlike ICER-based analyses, which evaluate the incremental cost-effectiveness of one strategy relative to another, NMB assesses the overall economic value of a strategy. Moreover, a strategy with the highest NMB at a given WTP threshold is generally considered economically preferable, particularly when multiple competing strategies are being compared and ICERs become more difficult to interpret (28, 29). When the analysis based on LYs was conducted using a WTP threshold of $41,859, both Ivon-Chem and Sint-Beva-Chem appeared potentially economically acceptable compared with chemotherapy. Furthermore, the ICER based on LYs for Sint-Beva-Chem compared with Ivon-Chem fell below this WTP threshold, suggesting that Sint-Beva-Chem may represent a potentially cost-effective option when evaluated using LYs. The use of LYs as an economic evaluation endpoint is also considered acceptable in oncology economic evaluations (30, 31). Although LYs do not capture quality-of-life differences and therefore should not be interpreted as definitive evidence of overall benefit, this finding suggests a potential advantage of Sint-Beva-Chem that warrants further investigation in future studies.

Additionally, a price adjustment analysis revealed that, if current drug prices were reduced, each regimen could become cost-effective or economically acceptable. In our analysis, Ivon-Chem remained economically acceptable for non-sqNSCLC following a price reduction in ivonescimab of approximately 16.9% or greater, indicating affordability under the current WTP threshold in China. When the price of ivonescimab was at approximately a 47.2% reduction, the regimen became cost-effective compared with chemotherapy, indicating that Ivon-Chem could provide greater health benefits at an acceptable additional cost. Similarly, the Sint-Beva-Chem regimen became cost-effective and economically acceptable after both sintilimab and bevacizumab were reduced by 59.3 and 37.5%, respectively. Likewise, the Atez-Beva-Chem regimen achieved cost-effectiveness and economically acceptable after both atezolizumab and bevacizumab were cut by 84.4 and 78.7%, respectively. A positive NMB indicates that a strategy provides value for money at a given WTP threshold, whereas ICER-based analyses determine whether the strategy is cost-effective relative to competing alternatives. Consequently, the price-reduction threshold identified by NMB-based analyses may be lower than that identified by ICER analyses. This finding highlights a major challenge in China’s healthcare system: how to control the soaring costs of cancer therapy. Policies such as centralized procurement, price negotiation, and value-based pricing are expected to substantially alleviate the financial burden on patients (32, 33). Effective cost control will facilitate broader clinical adoption of novel, beneficial treatment regimens and improve accessibility for patients with TKI-resistant non-sqNSCLC.

This study has several limitations. First, there were differences in the study populations across the three included RCTs. Whereas HARMONi-A and ORIENT-31 enrolled Chinese populations, the ATTLAS trial enrolled a multinational population. Due to the absence of head-to-head trial data, formal inconsistency testing could not be performed, which may have introduced potential bias. Second, the individual patient data applied in our model were algorithmically reconstructed rather than derived from actual patient-level datasets, which may have led to minor deviations from real-world data. However, we selected outcome reports with the longest available follow-up to minimize extrapolation-related uncertainty in the model. Third, the analysis included only costs and disutility associated with sAEs, while costs and disutility associated with grade 1–2 AEs were not included. Nevertheless, sensitivity analyses confirmed that the model results remained robust.

## 5 Conclusion

This study demonstrates that although Ivon-Chem, Sint-Beva-Chem, and Atez-Beva-Chem provide superior efficacy, they may also increase safety concerns. At the WTP threshold of $41,859/QALY, none of the three regimens was cost-effective compared with chemotherapy. Furthermore, substantial reductions in drug prices could improve their cost-effectiveness and economic acceptability.

## Data Availability

The original contributions presented in the study are included in the article/Supplementary material, further inquiries can be directed to the corresponding authors.

## Footnotes

1
https://automeris.io/wpd/

2
https://ypnew.hnsggzyjy.henan.gov.cn/
