# Supplementary material for: Cost-effectiveness analysis of combined immunotherapy regimens for non-squamous non-small lung cancer after tyrosine kinase inhibitors resistance
Source: Front Public Health. 2026 Jul 23;14:1860791. doi: 10.3389/fpubh.2026.1860791 (PMC13441919; doi:10.3389/fpubh.2026.1860791)
Supplement: Supplementary file 1 [file Supplementary_file_1.docx]

**Supplementary files**

**Supplementary tables**

**Table S1** The details of search in PubMed, Web of Science, Embase, and Cochrane library

**Table S2** Summary of Statistical Goodness-of-fit of Kaplan-Meier Curve

**Table S3** The CHEERS 2022 checklist

**Table S4** The details of including trails

**Table S5** The heterogeneity and publication bias analysis of all meta-analyses in the present study

**Table S6** The score of the surface under the cumulative ranking in effectiveness and safety

**Supplementary Figures**

**Figure S1** Overview of the study selection process

**Figure S2** The methodological quality and risk of bias of individual studies by the Cochrane Handbook

**Figure S3** Kaplan-Meier Curve according to reconstructed individual patient data

**Figure S4** AIC and BIC statistics for alternate parametric survival distributions

**Figure S5** Track in Markov model every cycle

**Figure S6** The surface under the cumulative ranking in effectiveness and safety

**Figure S7** Cost change in progression-free survival influence net benefit monetary

**Table S1 The details of search in PubMed, Web of Science, Embase, and Cochrane library**

| **Item** | **Query** |
| --- | --- |
| **#1** | Carcinoma, Non-Small-Cell Lung' OR 'Carcinoma, Non Small Cell Lung' OR 'Carcinomas, Non-Small-Cell Lung' OR 'Lung Carcinoma, Non-Small-Cell' OR 'Lung Carcinomas, Non-Small-Cell' OR 'Non-Small-Cell Lung Carcinomas' OR 'Carcinoma, Non-Small Cell Lung' OR 'Non-Small Cell Lung Cancer' OR 'Non-Small-Cell Lung Carcinoma' OR 'Non Small Cell Lung Carcinoma' OR 'Nonsmall Cell Lung Cancer' OR 'Non-Small Cell Lung Carcinoma' |
| **#2** | Immune Checkpoint Inhibitor' OR 'Checkpoint Inhibitors, Immune' OR 'Immune Checkpoint Blockers' OR 'Checkpoint Blockers, Immune' OR 'Immune Checkpoint Inhibitor' OR 'Checkpoint Inhibitor, Immune' OR 'PD-1 Inhibitors' OR 'PD 1 Inhibitors' OR 'Programmed Cell Death Protein 1 Inhibitor' OR 'Programmed Cell Death Protein 1 Inhibitors' OR 'PD-1 Inhibitor' OR 'Inhibitor, PD-1' OR 'PD 1 Inhibitor' OR 'Immune Checkpoint Blockade' OR 'Checkpoint Blockade, Immune' OR 'Immune Checkpoint Inhibition' OR 'Checkpoint Inhibition, Immune' OR 'PD-L1 Inhibitors' OR 'PD L1 Inhibitors' OR 'Programmed Death-Ligand 1 Inhibitors' OR 'Programmed Death Ligand 1 Inhibitors' OR 'PD-L1 Inhibitor' OR 'PD L1 Inhibitor' OR 'PD-1-PD-L1 Blockade' OR 'Blockade, PD-1-PD-L1' OR 'PD 1 PD L1 Blockade' |
| **#3** | pembrolizumab' OR 'MK-3475' OR 'Keytruda' OR 'lambrolizumab' OR 'SCH-900475' OR 'nivolumab' OR 'MDX-1106' OR 'Opdivo' OR 'BMS-936558' OR 'atezolizumab' OR 'MPDL-3280A' OR 'Tecentriq' OR 'camrelizumab' OR 'SHR-1210' OR 'carrelizumab' OR 'durvalumab' OR 'MEDI-4736' OR 'Imfinzi' OR 'tislelizumab ' OR 'tislelizumab-jsgr' OR 'BGB-A317' OR 'JHL-2108' OR 'tevimbra' OR 'toripalimab ' OR 'toripalimab-tpzi' OR 'sugemalimab ' OR 'Serplulimab' |
| **#4** | #2 OR #3 |
| **#5** | Bevacizumab' OR 'Avastin' OR 'Mvasi' OR 'Bevacizumab-awwb' OR 'Bevacizumab awwb' OR 'IBI305' OR 'bevacizumab biosimilar' |
| **#6** | #4 AND #5 |
| **#7** | Ivonescimab ' OR 'AK112' |
| **#8** | #6 OR #7 |
| **#9** | Clinical Trials, Randomized' OR 'Trials, Randomized Clinical' OR 'Controlled Clinical Trials, Randomized' OR 'randomized clinical trial ' OR 'RCT' OR 'clinical trial*' OR ' trial*' OR ' phase III' OR 'phase 3' OR 'clinical trial' |
| **#10** | #1 AND #8 #9 |

**Table S2 Summary of Statistical Goodness-of-fit of Kaplan-Meier Curve**

|  |  | Exponential | Weibull | Weibull (PH) | Log-normal | Log-logistic | Gamma | Gompertz | Generalized Gamma |
| --- | --- | --- | --- | --- | --- | --- | --- | --- | --- |
| ivon+chem | | |  |  |  |  |  |  |  |
| PFS | AIC | 746.8 | 719.0 | 719.0 | 703.0 | **702.6** | 711.5 | 738.3 | 704.4 |
|  | BIC | 749.9 | 725.2 | 725.2 | 709.2 | **708.8** | 717.6 | 744.5 | 713.6 |
| OS | AIC | 1005.5 | 998.4 | 998.4 | **979.0** | 981.0 | 993.5 | 1007.2 | 979.1 |
|  | BIC | 1008.6 | 1004.6 | 1004.6 | **985.2** | 987.2 | 999.6 | 1013.4 | 988.3 |
| atez+beva+chem | | |  |  |  |  |  |  |  |
| PFS | AIC | 1027.3 | 1004.1 | 1004.1 | **974.5** | 979.1 | 993.2 | 1023.1 | 971.7 |
|  | BIC | 1030.4 | 1010.2 | 1010.2 | **980.6** | 985.1 | 999.2 | 1029.2 | 980.8 |
| OS | AIC | 1016.5 | 1000.6 | 1000.6 | **990.5** | 991.6 | 996.5 | 1011.9 | 992.5 |
|  | BIC | 1019.5 | 1006.6 | 1006.6 | **996.5** | 997.6 | 1002.5 | 1017.9 | 1001.5 |
| sint+beva+chem | | |  |  |  |  |  |  |  |
| PFS | AIC | 693.5 | 674.3 | 674.3 | **667.4** | 667.8 | 670.2 | 687.0 | 669.0 |
|  | BIC | 696.6 | 680.4 | 680.4 | **673.6** | 673.9 | 676.4 | 693.1 | 678.2 |
| OS | AIC | 698.4 | 675.9 | 675.9 | 678.2 | 677.5 | **675.8** | 681.1 | 677.8 |
|  | BIC | 701.5 | 682.1 | 682.1 | 684.4 | 683.6 | **681.9** | 687.2 | 687.0 |
| chem | | |  |  |  |  |  |  |  |
| PFS | AIC | 1760.6 | 1728.6 | 1728.6 | **1672.0** | 1676.3 | 1708.8 | 1760.4 | 1671.6 |
|  | BIC | 1764.6 | 1736.6 | 1736.6 | **1680.0** | 1684.2 | 1716.8 | 1768.4 | 1683.5 |
| OS | AIC | 2192.8 | 2149.9 | 2149.9 | 2141.5 | **2139.6** | 2143.6 | 2172.7 | 2141.0 |
|  | BIC | 2196.8 | 2157.9 | 2157.9 | 2149.5 | **2147.5** | 2151.6 | 2180.7 | 2153.0 |

**Table S3 The CHEERS 2022 checklist**

| **Topic** | **No.** | **Item** | **Location where item is reported** |
| --- | --- | --- | --- |
| **Title** |  |  |  |
|  | 1 | Identify the study as an economic evaluation and specify the interventions being compared. | Title |
| **Abstract** |  |  |  |
|  | 2 | Provide a structured summary that highlights context, key methods, results, and alternative analyses. | Abstract |
| **Introduction** |  |  |  |
| **Background and objectives** | 3 | Give the context for the study, the study question, and its practical relevance for decision making in policy or practice. | Introduction |
| **Methods** |  |  |  |
| **Health economic analysis plan** | 4 | Indicate whether a health economic analysis plan was developed and where available. | Not applicable |
| **Study population** | 5 | Describe characteristics of the study population (such as age range, demographics, socioeconomic, or clinical characteristics). | Methods Section 2.3 |
| **Setting and location** | 6 | Provide relevant contextual information that may influence findings. | Methods Section 2.5 |
| **Comparators** | 7 | Describe the interventions or strategies being compared and why chosen. | Methods Section 2.5 |
| **Perspective** | 8 | State the perspective(s) adopted by the study and why chosen. | Methods Section 2.5 |
| **Time horizon** | 9 | State the time horizon for the study and why appropriate. | Methods Section 2.3 |
| **Discount rate** | 10 | Report the discount rate(s) and reason chosen. | Methods Section 2.5 |
| **Selection of outcomes** | 11 | Describe what outcomes were used as the measure(s) of benefit(s) and harm(s). | Methods Section 2.5 |
| **Measurement of outcomes** | 12 | Describe how outcomes used to capture benefit(s) and harm(s) were measured. | Methods Section 2.5 |
| **Valuation of outcomes** | 13 | Describe the population and methods used to measure and value outcomes. | Methods Section 2.5 |
| **Measurement and valuation of resources and costs** | 14 | Describe how costs were valued. | Not reported |
| **Currency, price date, and conversion** | 15 | Report the dates of the estimated resource quantities and unit costs, plus the currency and year of conversion. | Methods Section 2.5 |
| **Rationale and description of model** | 16 | If modelling is used, describe in detail and why used. Report if the model is publicly available and where it can be accessed. | Methods  Section 2.3 |
| **Analytics and assumptions** | 17 | Describe any methods for analysing or statistically transforming data, any extrapolation methods, and approaches for validating any model used. | Methods Section 2.4 and Supplementary files |
| **Characterising heterogeneity** | 18 | Describe any methods used for estimating how the results of the study vary for subgroups. | Not applicable |
| **Characterising distributional effects** | 19 | Describe how impacts are distributed across different individuals or adjustments made to reflect priority populations. | Methods Section 2.6 |
| **Characterising uncertainty** | 20 | Describe methods to characterise any sources of uncertainty in the analysis. | Methods Section 2.6 |
| **Approach to engagement with patients and others affected by the study** | 21 | Describe any approaches to engage patients or service recipients, the general public, communities, or stakeholders (such as clinicians or payers) in the design of the study. | Supplementary files |
| **Results** |  |  |  |
| **Study parameters** | 22 | Report all analytic inputs (such as values, ranges, references) including uncertainty or distributional assumptions. | Results Section 3.2 and Table 1 |
| **Summary of main results** | 23 | Report the mean values for the main categories of costs and outcomes of interest and summarise them in the most appropriate overall measure. | Results Section 3.2 |
| **Effect of uncertainty** | 24 | Describe how uncertainty about analytic judgments, inputs, or projections affect findings. Report the effect of choice of discount rate and time horizon, if applicable. | Results Section 3.3-3.4 |
| **Effect of engagement with patients and others affected by the study** | 25 | Report on any difference patient/service recipient, general public, community, or stakeholder involvement made to the approach or findings of the study | Not reported |
| **Discussion** |  |  |  |
| **Study findings, limitations, generalisability, and current knowledge** | 26 | Report key findings, limitations, ethical or equity considerations not captured, and how these could affect patients, policy, or practice. | Discussion |
| **Other relevant information** |  |  |  |
| **Source of funding** | 27 | Describe how the study was funded and any role of the funder in the identification, design, conduct, and reporting of the analysis | Section **Funding** |
| **Conflicts of interest** | 28 | Report authors conflicts of interest according to journal or International Committee of Medical Journal Editors requirements. | Section **Conflicts of interest** |

*From:* Husereau D, Drummond M, Augustovski F, et al. Consolidated Health Economic Evaluation Reporting Standards 2022 (CHEERS 2022) Explanation and Elaboration: A Report of the ISPOR CHEERS II Good Practices Task Force. Value Health 2022;25. <doi:10.1016/j.jval.2021.10.008>

**Table S4 The details of including trails**

| **Trials** | **ATTLAS, 2024** | | **HARMONi-A, 2024** | | **ORIENT-31, 2023** | |
| --- | --- | --- | --- | --- | --- | --- |
| **Non-small cell lung cancer** | sensitizing EGFR mutation or ALK translocation. All the patients experienced disease progression or intolerance to one or more EGFR or ALK TKIs | | EGFR variant whose disease had progressed after EGFR-TKIs therapy | | EGFR-mutated non-squamous NSCLC, disease progression after EGFR-TKIs treatment | |
| **Time** | August 27, 2019, and March 11, 2022 | | January 2022 to November 2022 | | July 11, 2019, and March 31, 2022 | |
| **Follow-up** | March 31, 2023 | | April, 2025 | | July, 4, 2022 | |
| **Country** | Korea | | China | | China | |
| **Design** | multicenter, open-label | | multicenter, double-blind | | multicenter, double-blind | |
| **Treatment (each 3-week cycle)** | | |  | |  | |
| induction treatment | atezolizumab (1200mg) plus bevacizumab (15mg/kg) with paclitaxel (175m²) and carboplatin (AUC,5 or 5.5 mg/mL/min) | pemetrexed (500 mg/m²) with carboplatin (AUC 5.0 mg/mL/min), or cisplatin (70 mg/m2) | ivonescimab (20 mg/kg) plus pemetrexed (500 mg/m²) with carboplatin (AUC 5 mg/mL/min) | pemetrexed (500 mg/m²) with carboplatin (AUC 5 mg/mL/min) | sintilimab (200 mg) plus IBI305 (15 mg/kg) with pemetrexed (500 mg/m²), and cisplatin (75 mg/m²) | pemetrexed (500 mg/m²) and cisplatin (75 mg/m²) |
| maintenance therapy | atezolizumab (1200 mg) plus bevacizumab (15 mg/kg) | pemetrexed (500mg/m^2^) | ivonescimab (20mg/kg) plus pemetrexed (500mg/m^2^) | pemetrexed (500 mg/m²) | sintilimab (200 mg) plus IBI305 (15 mg/kg) with pemetrexed (500 mg/m²) | pemetrexed (500 mg/m²) |
| **Patients (n)** | 151 | 74 | 161 | 161 | 158 | 160 |
| **PFS (95%CI)** |  |  |  |  |  |  |
| months | 8.48 (8,18-10.28) | 5.62 (4.27-7.20) | 7.1 (5.9-8.7) | 4.8 (4.2-5.6) | 7.2 (6.6-9.3) | 4.3 (4.1-5.3) |
| Hazard Ratio | 0.62 (0.45-0.86) |  | 0.46 (0.34-0.62) |  | 0.51 (0.39-0.67) |  |
| **OS (95%)** |  |  |  |  |  |  |
| months | 20.63 (18.14-15.59) | 20.27 (14.29-26.12) | 16.8 (14.5-20.0) | 14.1 (12.7-16.3) | 21.1 (17.5-23.9) | 19.2 (15.8-22.4) |
| Hazard Ratio | 1.01 (0.69-1.46) |  | 0.74 (0.58-0.95) |  | 0.98(0.72-1.34) |  |
| **Treatment-Related Adverse Events, No. (%)** | |  |  |  |  |  |
| Any | 146 (96.7) | 56 (75.7) | 160 (99.4) | 157 (97.5) | 156 (99%) | 155 (97%) |
| grade 3 or higher | 53 (35.1) | 11 (14.9) | 99 (61.5) | 79 (49.1) | 88 (56%) | 79 (49%) |

**Table S5 The heterogeneity and publication bias analysis of all meta-analyses in the present study**

|  | Heterogeneity | | Publication bias |
| --- | --- | --- | --- |
|  | P value for Q test | *I*^2^ | Egger's test |
| **progression-free survival** | 0.408 | 0.0% | 0.634 |
| **overall survival** | 0.245 | 0.3% | 0.251 |
| **adverse event** | 0.388 | 0.0% | 0.408 |
| **grade≥3 adverse event** | 0.127 | 0.5% | 0.248 |

**Table S6** **The score of surface under the cumulative ranking in effectiveness and safety**

|  | **overall survival** | **progression-free survival** | **adverse event** | **grade≥3 adverse event** |
| --- | --- | --- | --- | --- |
| Ivonescimab with chemotherapy | 0.9407 | 0.8663 | 0.5932 | 0.6154 |
| Sintilimab plus bevacizumab with chemotherapy | 0.3939 | 0.7088 | 0.4406 | 0.3689 |
| Atezolizumab plus bevacizumab with chemotherapy | 0.3393 | 0.4243 | 0.8853 | 0.9679 |
| chemotherapy | 0.3261 | 0.0006 | 0.0809 | 0.0478 |

**Figure S1 Overview of the study selection process**

**Figure S2 The methodological quality and risk of bias of individual studies by the Cochrane Handbook**

**Figure S3 Kaplan-Meier Curve according to reconstructed individual patient data**

(A-C) overall survival in IPC, ABPC, and SBPC compared with PC; (D-F) progression-free survival in IPC, ABPC, and SBPC compared with PC. IPC: ivonescimab with chemotherapy, PC: chemotherapy; ABPC, atezolizumab plus bevacizumab with chemotherapy; SBPC, sintilimab plus bevacizumab with chemotherapy.

**Figure S4 AIC and BIC statistics for alternate parametric survival distributions**

(A-D) overall survival in ivonescimab with chemotherapy, atezolizumab plus bevacizumab with chemotherapy, sintilimab plus bevacizumab with chemotherapy, and chemotherapy. (F-H) progression-free survival in ivonescimab with chemotherapy, atezolizumab plus bevacizumab with chemotherapy, sintilimab plus bevacizumab with chemotherapy, and chemotherapy. IPC, ivonescimab with chemotherapy; ABPC, atezolizumab plus bevacizumab with chemotherapy; SBPC, sintilimab plus bevacizumab with chemotherapy, PC, chemotherapy (pemetrexed with carboplatin or cisplatin).

**Figure S5 Track in Markov model every cycle**

(A) state in atez+beva+chem, ivon+chem, sint+beva+chem, and chem; (B) atez+beva+chem, ivon+chem, sint+beva+chem , and chem exhibiting in PFS, PD and death. ivon+chem, ivonescimab with chemotherapy; atez+beva+chem, atezolizumab plus bevacizumab with chemotherapy; sint+beva+chem, sintilimab plus bevacizumab with chemotherapy; chem, chemotherapy (pemetrexed with carboplatin or cisplatin); PFS, progression-free survival; PD, progress disease.

**Figure S6 The network analysis and surface under the cumulative ranking in effectiveness and safety**

(A) network plot of meta-analysis, (B) overall survival, (C) progression-free survival, (D) adverse events, and (E) grade 3 or higher adverse events. ivon+chem, ivonescimab with chemotherapy; atez+beva+chem, atezolizumab plus bevacizumab with chemotherapy; sint+beva+chem, sintilimab plus bevacizumab with chemotherapy; chem, chemotherapy (pemetrexed with carboplatin or cisplatin).

**Figure S7 Cost change in progression-free survival influence net benefit monetary**

ivon+chem, ivonescimab with chemotherapy; atez+beva+chem, atezolizumab plus bevacizumab with chemotherapy; sint+beva+chem, sintilimab plus bevacizumab with chemotherapy.
